# Cathepsin B as a potential serum biomarker for early diagnosis and progression of diabetic foot ulcer complicated with peripheral vascular disease

**DOI:** 10.1038/s41598-025-26599-5

**Published:** 2025-11-27

**Authors:** Ying Jin, Yaqi Yao, Yiqi Lin, Jianhua Zhong, Xinyi Kong, Yang Liu, Yuetong Li, Jie Qiao, Aixia Zhai, Changlong Bi

**Affiliations:** 1 Department of Endocrinology, The Eighth Affiliated Hospital, Sun Yat-sen University, No. 3025, Shennan Middle Road, Shenzhen, China; 2 Department of Laboratory Medicine, The Eighth Affiliated Hospital, Sun Yat-sen University, No. 3025, Shennan Middle Road, Shenzhen, China; 3 Biological Laboratory of Hetao Cooperation Zone, The Eighth Affiliated Hospital, Sun Yat-sen University, Shenzhen, China

**Keywords:** Diabetic foot ulcer, Peripheral vascular disease, Serum biomarker, Correlation analysis, Early diagnosis, Wagner grade, Biomarkers, Diseases

## Abstract

The existing diagnostic methods of diabetic foot ulcer (DFU) complicated with peripheral vascular disease (PVD) lack sufficient potential for early identification, which leads to slow wound healing, amputation and even death. Thus, this study aimed to explore the potential serum biomarkers of DFU complicated with PVD. A target gene of DFU complicated with PVD was identified using single-cell transcriptome analysis. The immunohistochemistry, ELISA, clinical correlation analysis, tubulogenesis assay, CCK8 assay, and scratch assay were used to verify the correlation between this target gene and DFU complicated with PVD. The ELISA experiment was used to detect the target gene in serum. In this study, the result of PPI in single-cell transcriptomes showed that cathepsin B (CTSB) was enriched in vascular endothelial cells of DFU. The immunohistochemistry and ELISA results revealed that CTSB was highly expressed in the tissues and serum of patients with the combination of DFU and PVD, and this expression increased with the increase of the Wagner grade of DFU. Clinical correlation analysis indicated that CTSB expression is positively correlated with the clinical indicators of the combination of DFU and PVD. Knockdown of CTSB promoted tubulogenesis, proliferation and migration of vascular endothelial cells and overexpression of CTSB has the opposite effect. CTSB, a secretory protein, can be detected as a diagnostic biomarker in serum. Therefore, this study suggested that CTSB can be used as a potential serum diagnostic biomarker for DFU complicated with PVD, which is helpful for the early diagnosis of this disease, prognosis monitoring and adjustment of treatment plans.

## Introduction

Diabetic foot ulcer (DFU) is a kind of foot infection, ulcer, or deep tissue destruction caused by distal limb neuropathy, peripheral vascular disease (PVD) and foot malformation in diabetic patients^1^. The prevalence of DFU in global patients with diabetes is estimated at 19%-34%, the number of DFU patients is increasing with the increasing prevalence of diabetes^2^. About 50% of DFU patients are accompanied by peripheral vascular disease according to statistics^3^. DFU complicated with PVD can lead to a series of hazards, including slow healing time of foot ulcers, lower limb amputation, subsequent cardiovascular events, and premature death^4^. Therefore, early identification of DFU combined with PVD enables targeted intervention measures to be initiated immediately, avoiding the further aggravation of the condition due to delayed diagnosis^5^.

At present, the diagnostic methods for DFU complicated with PVD include clinical examinations, ankle-brachial index (ABI) determination, color Doppler ultrasound (CDU), computed tomography angiography (CTA), and magnetic resonance angiography (MRA)[4]. However, there still exist limitations in the diagnostic methods for DFU complicated with PVD. The clinical examinations for determining vascular patency of the foot pulse by clinicians have limited accuracy^6^. Moreover, the ABI method may present false positive results in DFU patients with calcification of the artery wall^6^. On the other hand, the CDU method has high technical requirements for the examiner, and its ability to detect deep blood vessels and some small blood vessel lesions is limited^7^. Besides, the CTA method requires the use of iodine-containing contrast media, which may cause allergic reactions or pose a risk of damage to kidney function in DFU patients^6^. For the MRA method, there is a long image acquisition time in need and cannot be used in patients with incompatible ferromagnetic devices^6^. Currently, potential serum biomarkers with the advantages of easy sampling, low trauma, potentiality, and rapidity are potentially valuable early detection methods for diseases^8^. Although some serum biomarkers, such as vascular endothelial growth factor (VEGF), angiopoietins, and endothelial microparticles, have been suggested to be associated with DFU complicated with PVD, the potentiality and sensitivity of these markers are not ideal^9^.

Notably, cathepsin B (CTSB), a lysosomal cysteine protease with both endopeptidase and exopeptidase activities, has been linked to vascular pathophysiological processes^10^. It participates in extracellular matrix remodeling, inflammatory responses, and angiogenesis regulation, which are the key processes involved in the development and progression of PVD^11^. As a secretory protein, CTSB can be detected in serum, making it a potential candidate for non-invasive biomarker screening^10^. However, its role as a serum biomarker for DFU complicated with PVD remains unclarified. Therefore, there is an urgent need to explore the potential of CTSB as a diagnostic biomarker for DFU complicated with PVD, to facilitate early diagnosis, real-time monitoring of disease progression and prognosis, and optimization of treatment plans.

## Methodology

### Ethics approval

Ethical approval for this study was granted by the local research ethics committee of the Eighth Affiliated Hospital of Sun Yat-sen University (No.2025-030-2). The clinical blood and tissue samples were all derived from the residual specimens after clinical diagnosis and treatment of patients at the Eighth Affiliated Hospital of Sun Yat-sen University. The clinical indicators of patients were gathered from the Eighth Affiliated Hospital of Sun Yat-sen University’s electronic medical record system. The experiments involving human participants had obtained the informed consent of the patients. The amended Declaration of Helsinki was followed while conducting medical research on humans.

### Single-cell transcriptome

The single-cell transcriptome data were downloaded from the Gene Expression Omnibus (GEO) databases (GSE165816 and GSE223964). Among them, foot ulcer skin samples from 5 diabetic patients were obtained from GSE165816, and foot ulcer skin samples from 4 diabetic patients and 4 non-diabetic subjects were derived from GSE223964. This study employed the official Space Ranger software (10×Genomics) to process the raw sequencing data in FASTQ format for cell barcode identification, unique molecular identification, quantitative gene expression analysis, and basic data preprocessing. STAR comparison software was utilized for genome reference comparison, with Cell Ranger hg38 serving as the reference genome. Sample quality control and subsequent data normalization were conducted using STutility software (v1.10.0). Cells were filtered based on the following criteria: number of detected genes between 200 and 6,000, UMI counts < 20,000, and mitochondrial gene content < 10% to exclude low-quality cells and potential doublets. Batch effects between samples were corrected using the Harmony algorithm implemented in STutility. Vascular endothelial cell clusters were identified and annotated based on the expression of canonical vascular endothelial markers. Differential gene expression analysis between DFU and non-DFU vascular endothelial cells was performed using the cluster Profiler software package (v4.6.0) with the Wilcoxon rank-sum test. Genes were considered significantly differentially expressed with thresholds set at adjusted p-value < 0.05 and absolute log2 fold change > 1. Among the down-regulated genes enriched in DFU, the combinations of pixels per inch (PPI) analysis and EcCentricity method of single-cell transcriptome were used to calculate the scores of the correlated degree between genes, to further select the genes with the highest correlated degree in DFU. This integrated approach was used to identify genes with the highest correlation and potential functional significance in DFU pathogenesis.

### Detection of the CTSB expression in tissues by using immunohistochemistry

Tissues were collected from patients with and without the combination of DFU and PVD. Among them, the DFU samples are classified into Wagner grade 3, Wagner grade 4 and Wagner grade 5. The tissues were immediately fixed in 10% neutral-buffered formalin at room temperature. After fixation, they were dehydrated in graded alcohols, cleared in xylene, and embedded in paraffin wax. Paraffin-embedded blocks were sectioned into 3 µM thick slices using a microtome. The sections were floated on a water bath, transferred onto positively charged slides, air-dried overnight, and stored in a desiccator. Slides were dewaxed in xylene and rehydrated through a series of graded alcohols and distilled water. Sections were blocked with 10% normal serum at room temperature for 2 h. The primary antibody, diluted in a buffer, was applied to the sections and incubated overnight. After three 5-minute washes with PBS, the secondary antibody, conjugated to a detectable label, was added and incubated at room temperature for 2 hours in the dark. Another three 5-minute PBS washes were carried out. For peroxidase-conjugated secondary antibodies, 3,3’ - diaminobenzidine was used for chromogenic detection. For chromogenic immunohistochemistry, hematoxylin counterstaining was done, followed by dehydration, clearing, and mounting with Permount. Stained slides were examined under a microscope, images were captured, and image analysis software was used for quantification.

### Detection of the CTSB content in serum using ELISA assay

Serum was collected from patients with and without the combination of DFU and PVD. Among them, the DFU patients had different Wagner grades from grade 3 to grade 5. The CTSB ELISA assay was used to detect the serum of the samples. The 100 µL of standards and cell culture supernatants were then added to the ELISA microplate after the standards had been serially diluted. The fluid in the microplate’s well was disposed of after the ELISA microplate had been incubated for 90 min at 37 °C. After adding 100 µL of biological antibody/antigen working solution to the microplate, it was incubated for 60 min at 37 °C. After discarding the liquid, the microplate was cleaned three times using a washing buffer. After that, 100 µL of the enzyme-conjugate working solution was added to the microplate, and it was incubated for 30 min at 37 °C. After discarding the liquid, the microplate was cleaned five times using a washing buffer. For the color-developing reaction, 90 µL of the substrate solution was added to the microplate, and it was then incubated for 15 min at 37 °C. To stop the color-developing reaction in the microplate, 50 µL of the stop solution was applied. A microplate reader was used to measure the absorbance at 450 nm, and the standard curve was used to determine the sample’s concentration.

### Clinical correlation analysis of CTSB expression

The clinical correlation analysis between CTSB expression and clinical indicators of patients with the combination of DFU and PVD was carried out. The clinical indicators in the clinical correlation analysis include gender, BMI, heart rate, DBP, cigarette smoking, hypertension history, dyslipidemia history, nonhemorrhagic stroke history, peripheral arterial disease history, diabetes mellitus history, total cholesterol, triglyceride, LDL-C, HDL-C, platelet counts, high blood glucose and leukocyte count.

### Verification of angiogenesis function of CTSB in vitro

#### Cell culture and sub-culturing

Human umbilical vein endothelial cells (HUVECs) were purchased from the American Type Culture Collection (ATCC). Cells were cultured in Dulbecco`s Modified Eagle Medium (DMEM) supplemented with 10% fetal bovine serum (FBS) and 1% penicillin-streptomycin and maintained in a humidified incubator at 37 °C with 5% CO2.

For sub-culturing, when cells reached 80%–90% confluence, medium was aspirated and cells were rinsed twice with phosphate-buffered saline (PBS). 0.25% trypsin-EDTA was added for 6 min at 37 °C to detach cells, with digestion terminated by adding DMEM with FBS. Cell suspensions were centrifuged and the pellet was resuspended in fresh medium and seeded into new flasks at a 1:3 dilution. After 3 times passages, the cells exhibited normal spindle-shaped morphology, and were then used for subsequent experiments.

#### Establishment of CTSB interference model in HUVECs

The gene sequences of CTSB were synthesized and cloned into an expression vector (pEGFP-N1) to be designed as an overexpression interference model. The construction of the overexpression plasmids was completed by the MiaoLing Plasmid Platform (Wuhan, China), and the vector was verified by sequencing. The siRNA of CTSB was designed to be a knockdown interference model and synthesized by Genepharma (Suzhou, China) and its sequences can be found in Table S1. The CTSB plasmids, CTSB siRNA, and the combination of CTSB plasmid with CTSB siRNA were transfected into HUVECs using Lipofectamine 2000 transfection reagent (Invitrogen, 11668019) according to the manufacturer’s protocol, to establish the CTSB interference model in HUVECs.

#### The angiogenesis effect of CTSB on the HUVECs using the tubule formation method

CTSB expression was modulated in HUVECs via knockdown, overexpression, or co-transfection (transfection with CTSB plasmid and CTSB siRNA). The angiogenesis of HUVECs was subsequently detected by using a tubule formation method. The matrigel was added to the 24-well plate and solidified in the cell incubator for 60 min. The cell suspension of HUVECs was then inoculated into the 24-well plate at a density of 2 × 10^5^ cells/mL. The cells and matrigel were incubated at 37℃ for 6 h, and the formation of tubes was observed and photographed under the microscope. The number and length of branches were measured and analyzed statistically by ImageJ software. The data results were expressed as three independent experiments.

#### The proliferation effect of CTSB on the HUVECs using cell counting kit-8 (CCK-8)

CTSB expression in HUVECs was modulated through knockdown, overexpression, or co-transfection approaches, and the proliferation ability of HUVECs during angiogenesis was detected by CCK8. The cells seeded at a density of 5000 cells per well were inoculated on 96-well plates and cultured in a 5% CO_2_ incubator at 37℃. At the time points of 0 h, 12 h, 24 h, 36 h and 48 h after seeding, the cells were incubated with 5 mg/mL/well CCK8 solution for 2 h. The optical density was then detected with a microplate reader at 450 nm. The data results were expressed as the mean SD of three independent experiments.

#### The migration effect of CTSB on the HUVECs using scratch assay

Knockdown, overexpression, and co-transfection were used to modulate CTSB expression in HUVECs, and the migration ability of HUVECs during angiogenesis was detected by scratch assay. Briefly, cells were cultured in 6-well plates until reaching confluence and then were scratched with a 200 µL pipette tip. The cells were cultured in an incubator, followed by observing gap widths at 0 h, 12 h and 24 h, as well as photographing under a microscope. ImageJ software was used to measure the gap widths. The relative migration rate was calculated and the data results were expressed as the mean SD of three independent experiments.

### Statistical analysis

GraphPad Prism 9 (GraphPad, USA) was used to conduct the statistical analyses. All data are presented as mean ± standard deviation unless otherwise specified. The sample size (n) for each experiment represents the number of independent biological replicates, which is indicated in the figure legends. One-way ANOVA was used to evaluate multiple comparisons, followed by Bonferroni correction for statistical hypothesis testing to account for multiple comparisons. Correlation analysis was conducted using Pearson’s correlation coefficient to evaluate the relationship between CTSB expression levels and clinical indicators. A p-value of less than 0.05 was considered statistically significant.

## Results

### CTSB as a potential target gene for DFU complicated with PVD

**Fig. 1:**
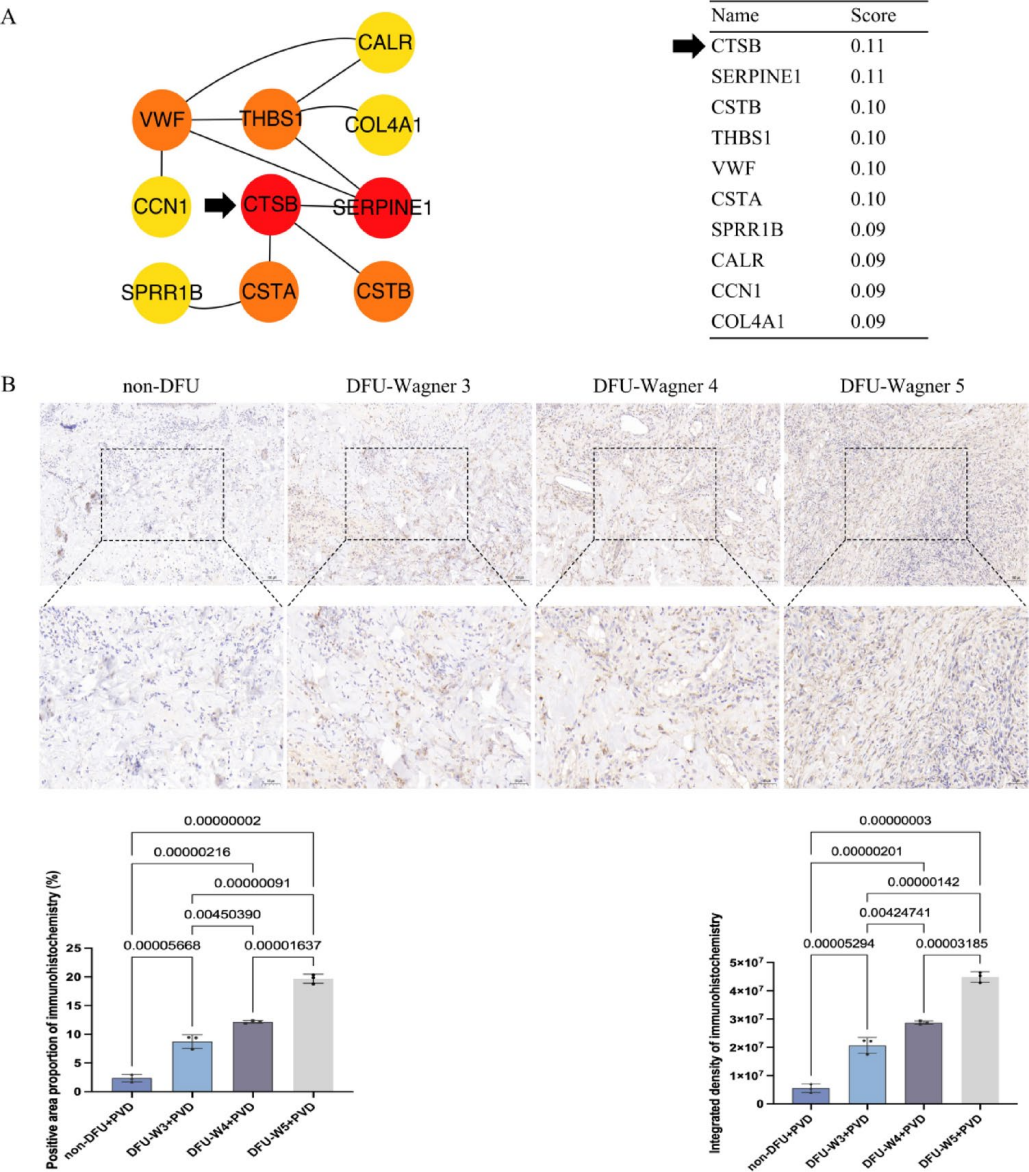
CTSB is a potential target gene for patients with the combination of DFU and PVD.

For the analysis of cell clusters, vascular endothelial cells were analyzed, as these cells are critical for angiogenesis and vascular homeostasis^12^. Vascular endothelial cells dynamically regulate blood vessel formation, permeability, and nutrient exchange, making them pivotal in wound healing processes disrupted in DFU^13^. In this study, while comparing vascular endothelial cells between DFU and non-DFU samples, CTSB was identified as a significantly differentially expressed gene. CTSB expression was notably downregulated in DFU vascular endothelial cells, with a log_2_ fold change (log_2_FC) of – 0.3505 and a p-value of 5.87 × 10^− 36^. This met the pre-defined thresholds for significant differential expression (adjusted p value < 0.05 and log_2_FC > 1). To further explore the functional relevance of CTSB in DFU, we analyzed PPI networks. Using differentially expressed genes as inputs, we constructed a PPI network via the STRING database (setting a confidence score threshold > 0.7 to ensure network reliability. Subsequent application of the EcCentricity algorithm to this network prioritized genes by their topological centrality, and CTSB emerged as a top hub gene (Fig. 1, A). This result suggested CTSB is a potential target gene for DFU complicated with PVD.

A represents the gene connectivity in the PPI of the single-cell transcriptome. PPI network was first constructed using differentially expressed genes in endothelial clusters via STRING. The EcCentricity algorithm was then applied to calculate node centrality, prioritizing hub genes with high connectivity and functional relevance. CTSB was identified as a top candidate due to its high EcCentricity score and direct interactions with key angiogenesis-related genes. B represents the CTSB expression in immunohistochemistry of clinical samples. Panels show low-magnification views and the dashed boxes indicate regions enlarged in bottom panels. Quantification includes positive area proportion and integrated density. non-DFU + PVD: non-diabetic foot ulcer combined with peripheral vascular disease (*n* = 3); DFU-W3 + PVD: Wagner grade 3 diabetic foot ulcer combined with peripheral vascular disease (*n* = 3); DFU-W4 + PVD: Wagner grade 4 diabetic foot ulcer combined with peripheral vascular disease (*n* = 3); DFU-W5 + PVD: Wagner grade 5 diabetic foot ulcer combined with peripheral vascular disease (*n* = 3).

### Clinical correlations between CTSB and DFU combined with PVD

#### Immunohistochemistry result revealed clinical correlation

Since CTSB in vascular endothelial cells is a potential target gene for DFU combined with PVD, this study intends to evaluate the clinical correlation between CTSB and DFU combined with PVD in tissue samples through immunohistochemistry. From the results of positive area and integrated density of immunohistochemistry, the expression of CTSB in tissue samples of patients with the combination of DFU and PVD was higher than that in patients without the combination of DFU and PVD (Fig. 1, B) (Table S2). Furthermore, the expression level of CTSB increased with the increase of the Wagner grade of DFU tissue samples in the immunohistochemistry result (Fig. 1, B) (Table S2). This result suggested that the expression of CTSB is clinically related to DFU complicated with PVD in tissue samples.

#### ELISA result revealed clinical correlation

Since CTSB in vascular endothelial cells was found to be a potential target gene for DFU complicated with PVD, this study intended to further evaluate the clinical correlation between CTSB and DFU combined with PVD through ELISA. In the baseline of sample selection, there were significant differences in the peripheral arterial disease history, diabetes mellitus history, blood glucose and leukocyte count between patients with DFU and PVD combination and those without DFU and PVD combination (Table 1). This is because patients with the combination of DFU and PVD normally have peripheral arterial disease history, diabetes mellitus history, high blood glucose and leukocyte count caused by inflammation. On the contrary, the remaining indicators, including gender, BMI, heart rate, DBP, cigarette smoking, hypertension, dyslipidemia, no hemorrhagic stroke, total cholesterol, triglyceride, LDL-C, HDL-C and platelet counts, showed no significant differences among the various groups in the baseline of sample selection (Table 1).

**Table 1:** Characteristics of patients with and without the combination of DFU and PVD at baseline.

Characteristics		non-DFU + PVD (*n* = 36)	DFU-W3 + PVD (*n* = 26)	DFU-W4 + PVD (*n* = 21)	DFU-W5 + PVD (*n* = 10)	*P* value				
						non-DFU+PVD vs DFU-W3+PVD	non-DFU+PVD vs DFU-W4+PVD	non-DFU+PVD vs DFU-W5+PVD	DFU-W3+PVD vs DFU-W4+PVD	DFU-W4+PVD vs DFU-W5+PVD
Demographics										
Women	n (%)	19.00 (52.78)	9.00 (34.62)	11.00 (52.38)	2.00 (20.00)	0.48	> 0.9999	0.2540	0.6118	0.8563
BMI	kg/m2	25.19 (4.49)	29.49 (15.52)	22.02 (2.18)	21.65 (2.54)	0.33	0.9798	0.9799	0.1558	0.3197
Clinical features										
Heart rate	bpm	81.08 (11.53)	89.62 (14.4)	86.38 (15.19)	85.90 (9.80)	0.0626	0.5063	0.7647	0.6438	0.7380
DBP	mmHg	80.56 (17.63)	80.50 (6.59)	82.29 (12.10)	83.90 (13.87)	> 0.9999	0.9673	0.9027	0.9703	0.9086
Cigarette smoking	n (%)	5.00 (13.89)	8.00 (30.77)	6.00 (28.57)	3.00 (30.00)	0.42	0.5956	0.7173	0.9981	> 0.9999
Medical history										
Hypertension	n (%)	7.00 (19.44)	16.00 (61.54)	16.00 (76.19)	6.00 (60.00)	0.0026*	< 0.0001*	0.0632	0.6841	0.9997
Dyslipidemia	n (%)	13.00 (36.11)	5.00 (19.23)	2.00 (9.52)	0.00 (0.00)	0.3592	0.0791	0.0616	0.8403	0.5679
Nonhemorrhagic stroke	n (%)	1.00 (2.78)	0.00 (0.00)	2.00 (9.52)	0.00 (0.00)	0.9285	0.5091	0.9714	0.2635	> 0.9999
Peripheral arterial disease	n (%)	5.00 (13.89)	19.00 (73.08)	16.00 (76.19)	8.00 (80.00)	< 0.0001*	< 0.0001*	0.0002*	0.9941	0.9697
Diabetes mellitus	n (%)	0.00 (0.00)	26.00 (100.00)	21.00 (100.00)	10.00 (100.00)	< 0.0001*	< 0.0001*	< 0.0001*	> 0.9999	> 0.9999
Biomarkers										
Total cholesterol	mmol/L	4.58 (1.02)	4.59 (1.20)	3.91 (1.13)	4.62 (1.35)	> 0.9999	0.1819	0.9998	0.2270	> 0.9999
Triglyceride	mmol/L	1.71 (1.80)	1.47 (1.79)	1.17 (0.39)	1.08 (0.50)	0.9334	0.6124	0.6568	0.9196	0.9035
LDL-C	mmol/L	2.87 (0.67)	2.93 (0.89)	2.45 (0.85)	2.98 (1.03)	0.9929	0.3085	0.9802	0.2527	0.9980
HDL-C	mmol/L	1.11 (0.32)	0.99 (0.23)	0.98 (0.39)	1.05 (0.34)	0.4757	0.4462	0.9338	0.9985	0.9683
Platelet counts	109/L	271.85 (89.34)	269.15 (108.00)	316.00 (89.87)	306.30 (118.15)	0.9996	0.3747	0.7650	0.3713	0.7413
Blood glucose	mmol/L	5.08 (0.80)	9.84 (5.24)	9.24 (4.06)	9.28 (2.83)	< 0.0001*	0.0003*	0.0075*	0.9398	0.9739
Leukocyte count	109/L	6.34 (1.46)	8.90 (2.97)	11.01 (3.72)	9.13 (3.10)	0.0031*	< 0.0001*	0.0290*	0.0504	0.9959

This table characterizes baseline differences in patient cohorts, highlighting clinical factors associated with DFU complicated with PVD. P values compare differences across groups, with * denoting statistical significance set at *p* < 0.05. non-DFU + PVD: non-diabetic foot ulcer combined with peripheral vascular disease (*n* = 36); DFU-W3 + PVD: Wagner grade 3 diabetic foot ulcer combined with peripheral vascular disease (*n* = 26); DFU-W4 + PVD: Wagner grade 4 diabetic foot ulcer combined with peripheral vascular disease (*n* = 21); DFU-W5 + PVD: Wagner grade 5 diabetic foot ulcer combined with peripheral vascular disease (*n* = 10).

**Fig. 2:**
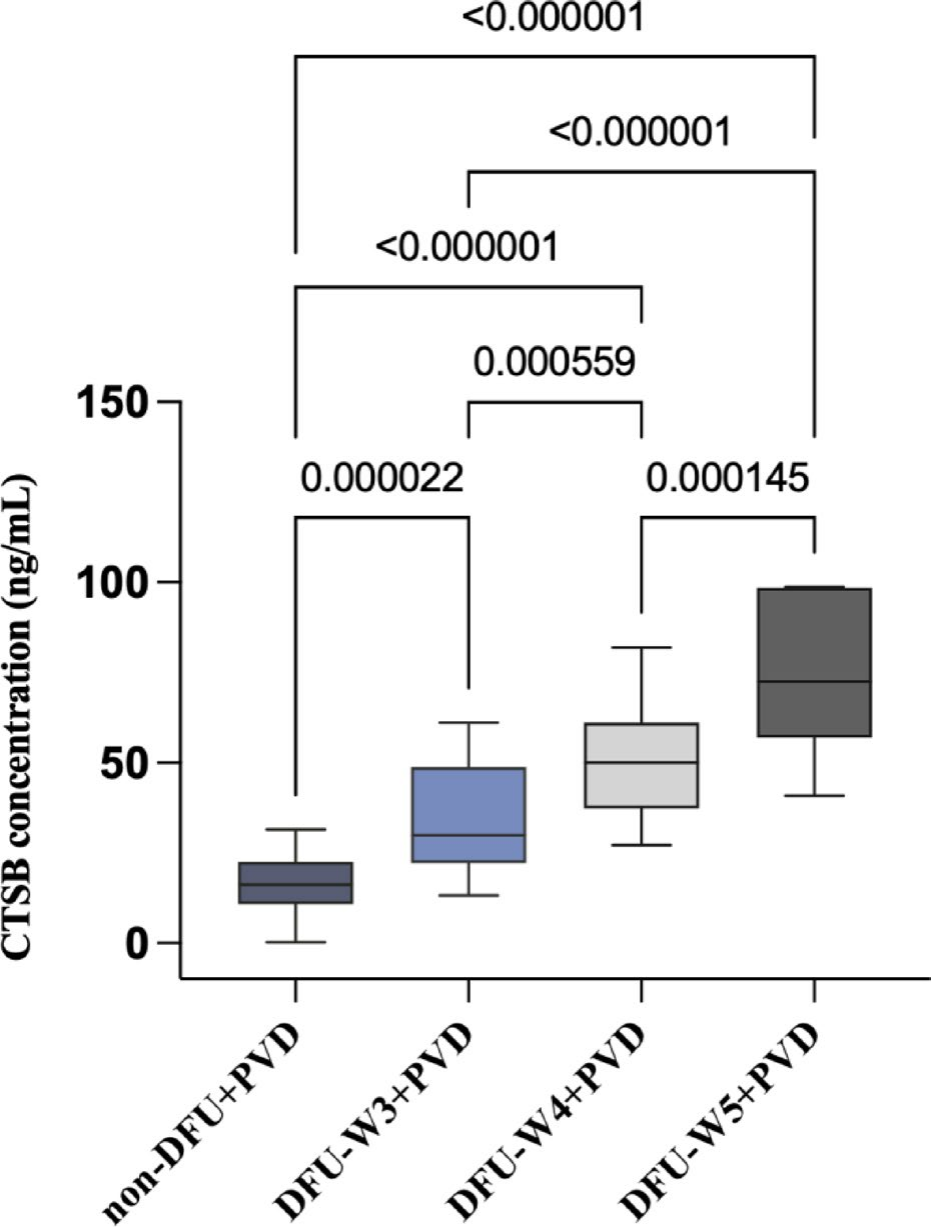
The expression of CTSB in the serum of patients with and without the combination of DFU and PVD.

Based on the ELISA result, the CTSB expression level was higher in serum samples of patients with the combination of DFU and PVD than in serum samples of patients without the combination of DFU and PVD (Fig. 2) (Table S3). ELISA results also revealed that CTSB expression level increased with the increase of DFU Wagner grade (Fig. 2) (Table S3). This result suggested that the expression of CTSB was clinically related to DFU complicated with PVD in serum samples.

Box and whisker plots depict CTSB concentrations (pg/mL) in serum from clinical samples. Whiskers represent minimum or maximum values, boxes show interquartile ranges, and centers mark medians. P values above brackets denote statistical differences between groups. non-DFU + PVD: non-diabetic foot ulcer combined with peripheral vascular disease (*n* = 36); DFU-W3 + PVD: Wagner grade 3 diabetic foot ulcer combined with peripheral vascular disease (*n* = 26); DFU-W4 + PVD: Wagner grade 4 diabetic foot ulcer combined with peripheral vascular disease (*n* = 21); DFU-W5 + PVD: Wagner grade 5 diabetic foot ulcer combined with peripheral vascular disease (*n* = 10).

#### The clinical correlation between CTSB and DFU complicated with PVD

**Fig. 3:**
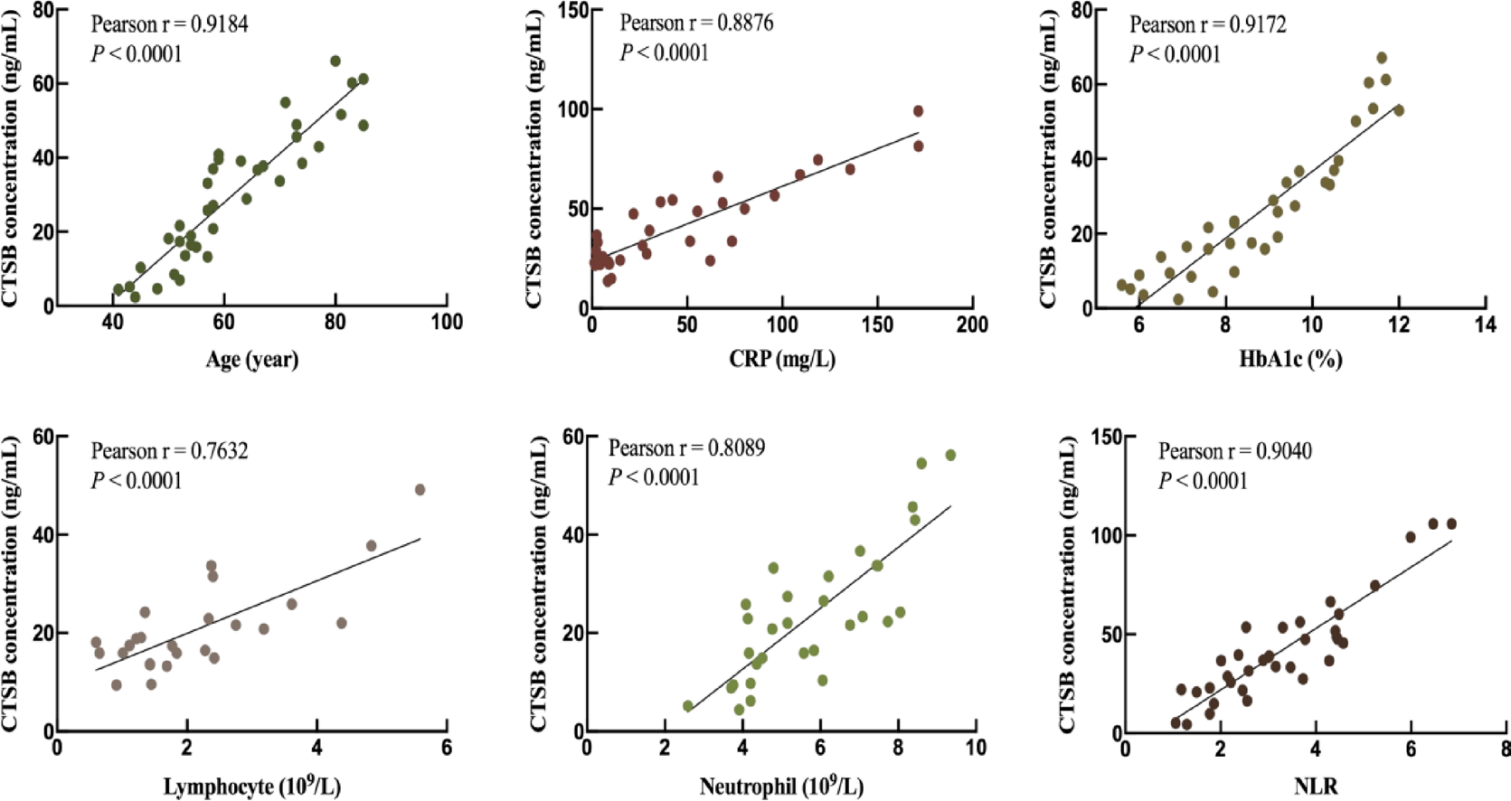
Correlation analysis of serum CTSB expression and clinical indicators of DFU complicated with PVD.

Since CTSB in vascular endothelial cells was found to be a potential target gene for DFU complicated with PVD, a clinical correlation analysis between CTSB expression and clinical indicators of patients with the combination of DFU and PVD was performed. Based on the previous study, the clinical indicators of age, C-reactive protein (CRP), Hemoglobin A1c (HbA1c), lymphocyte, neutrophil and Neutrophil-to-lymphocyte ratio (NLR), were reported to be the risk factors of DFU complicated with PVD^14,15^. In this study, the Pearson correlation coefficient (r) between age and serum CTSB expression was 0.9184, while the Pearson correlation coefficient (r) between CRP and serum CTSB expression was 0.8876 (Fig. 3). Between HbA1c and serum CTSB expression, the Pearson correlation coefficient (r) was 0.9172 (Fig. 3). Moreover, the Pearson correlation coefficients (r) were 0.7632 for lymphocyte versus serum CTSB expression, as well as 0.8089 for neutrophil versus serum CTSB expression (Fig. 3). Meanwhile, for the relationship between NLR and serum CTSB expression, the Pearson correlation coefficient (r) reached 0.9040 (Fig. 3). The Pearson correlation coefficients (r) of these clinical indicators were all greater than 0.7, and the P values were less than 0.0001, indicating that they have a positive clinical correlation between serum CTSB expression and clinical indicators of DFU complicated with PVD (Fig. 3).

Scatter plots illustrate Pearson correlations between serum CTSB concentrations (pg/mL) and clinical indicators in patients with DFU complicated by PVD: age (*n* = 37); CRP (*n* = 32); hbA1c (*n* = 35); lymphocyte (*n* = 25); neutrophil (*n* = 31); NLR (*n* = 35). CRP: c-reactive protein; HbA1c: hemoglobin A1c; NLR: neutrophil-to-lymphocyte ratio.

#### Tube formation, proliferation, and migration assays revealed clinical correlation

**Fig. 4:**
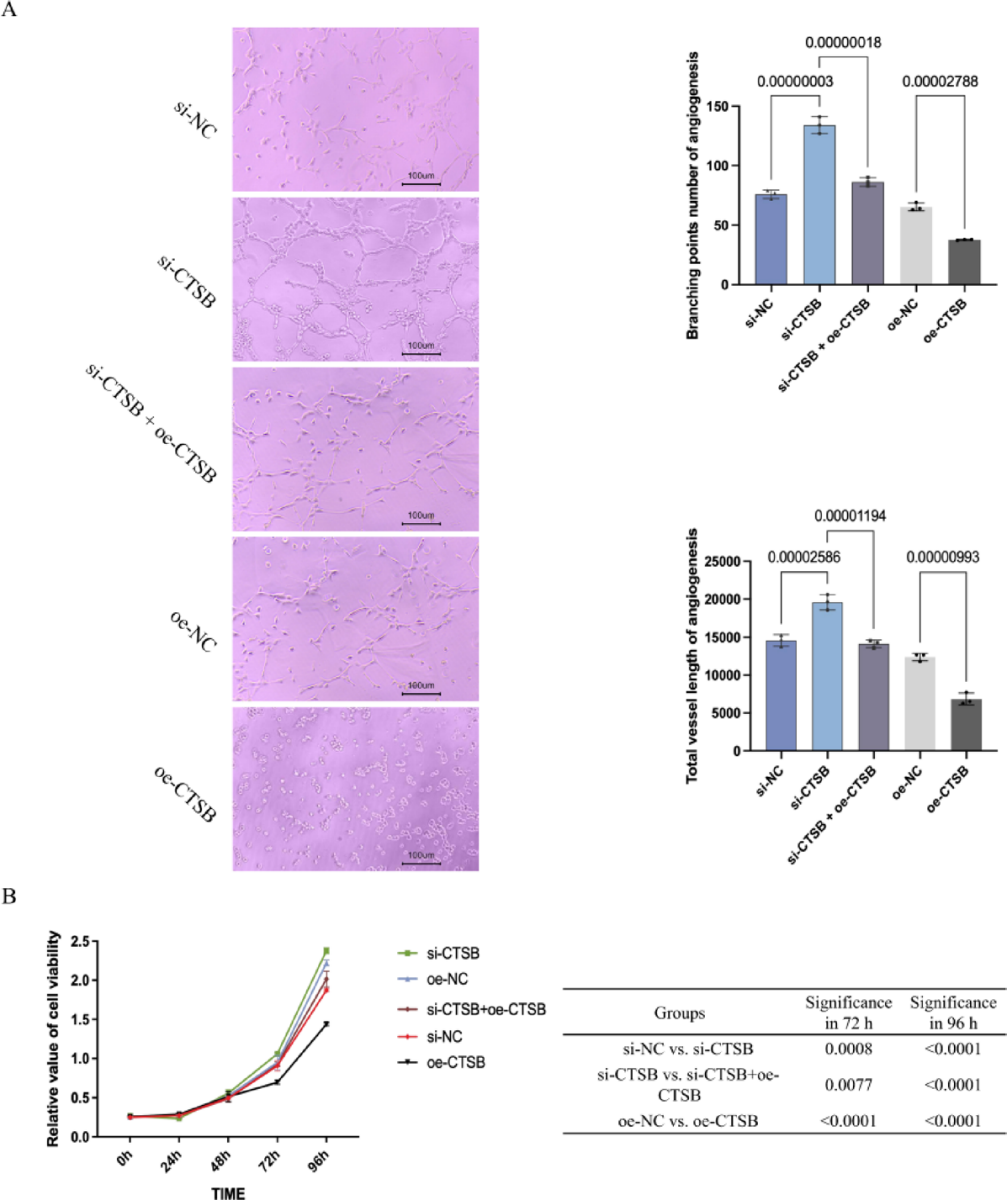
The influence of CTSB on angiogenesis and proliferation in vascular endothelial cells.

The effect of CTSB on angiogenesis (tube formation, proliferation, migration) was also detected to verify the clinical correlation between CTSB and DFU combined with PVD. In the tube formation assay, knockdown of CTSB was found to promote the branching points and total vessel length of vascular endothelial cells, while overexpression of CTSB had the opposite effect (Fig. 4, A). Co-transfection of CTSB knockdown and overexpression was found to have a saving effect on the branching points and total vessel length of vascular endothelial cells (Fig. 4, B).

The results of the CCK8 assay demonstrated that the knockdown of CTSB significantly promoted the proliferation of vascular endothelial cells (Fig. 4). Conversely, overexpression of CTSB exerted an inhibitory effect on the proliferation of these cells, as illustrated in Fig. 4. Notably, the co-transfection of CTSB was observed to have a compensatory effect on the proliferation of vascular endothelial cells, as depicted in Fig. 4.

**Fig. 5:**
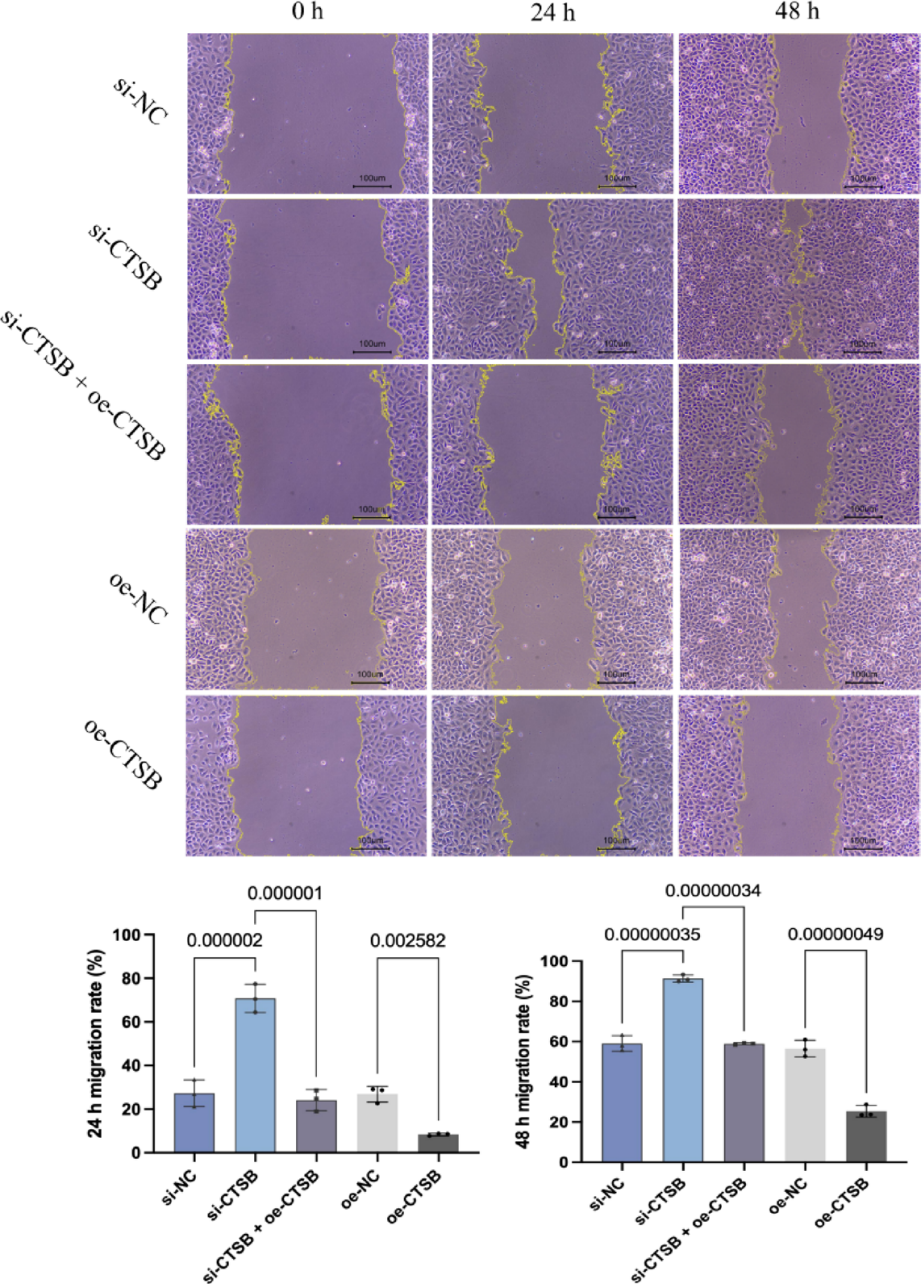
The impact of CTSB on the migration of vascular endothelial cells.

The scratch assay result demonstrated that the migration capacity of HUVECs was delayed, promoted, and saved by CTSB overexpression, knockdown and co-transfection, respectively, according to the 24 h and 48 h migration rate (Fig. 5). Therefore, the results of tube formation assay, CCK8 assay and scratch assay indicated that the reduction of CTSB had an adverse effect on angiogenesis in DFU, suggesting that CTSB is associated with DFU complicated with PVD.

A represents the influence of CTSB on angiogenesis in vascular endothelial cells; Images show tubule generation situation; Bar graphs quantify the branching points number of angiogenesis and total vessel length of angiogenesis. B represents the influence of CTSB on proliferation in vascular endothelial cells. Data are presented as means ± standard deviation of three independent experiments. si-NC: knockdown control; si-CTSB: CTSB knockdown; si-CTSB + oe-CTSB: CTSB knockout and overexpression co-transfection; oe-NC: overexpression control; oe-CTSB: CTSB overexpression.

Images show migration at 0 h, 24 h and 48 h. Bar graphs quantify 24 h and 48 h migration rates. Data are presented as means ± standard deviation of three independent experiments. si-NC: knockdown control; si-CTSB: CTSB knockdown; si-CTSB + oe-CTSB: CTSB knockout and overexpression co-transfection; oe-NC: overexpression control; oe-CTSB: CTSB overexpression.

## Discussion

CTSB, a secretory protease with dual functions in regulating pathophysiological processes, has shown potential as a serum biomarker in various diseases. In patients with nasopharyngeal carcinoma, a previous study found that serum CTSB levels are closely associated with tumor stage and lymph node metastasis, consistent with its role in extracellular matrix remodeling and tumor invasion^16^. In rheumatoid arthritis, research demonstrated that serum CTSB levels increase with disease activity, as it mediates synovial inflammation and cartilage degradation through protease activation^17^. Importantly, in cardiovascular diseases such as atherosclerosis, it was confirmed that CTSB is related to arterial stiffness and plaque instability, with serum levels reflecting the severity of peripheral arterial dysfunction^11^. This pathological feature is highly consistent with PVD, the focus of this study. These precedents indicate that CTSB has potential as a serum biomarker in diseases involving vascular dysfunction or tissue damage. Additionally, the secretory nature of CTSB, confirmed by ELISA results, further supports its advantage as a non-invasive biomarker. Unlike tissue markers, serum CTSB can be repeatedly detected to monitor disease progression or treatment response, which is of great guiding value for clinical decision-making in DFU.

The clinical significance of CTSB as a serum biomarker for DFU complicated with PVD is further supported by its association with known disease indicators. Correlation analysis in this study showed that serum CTSB levels were positively correlated with inflammatory markers (CRP and NLR) and indicators of poor glycemic control (HbA1c), these indicators have been confirmed to promote DFU progression^9^. Notably, this study found that serum CTSB levels increased with Wagner grade, consistent with the severity of ulceration and underlying vascular damage. This is consistent with observations in other vascular diseases, such as in chronic venous insufficiency, where serum levels of proteases involved in angiogenesis are associated with wound healing outcomes^7^.

The clinical correlation between CTSB and DFU complicated with PVD stems from its multi-dimensional regulation of vascular endothelial cell function, a core process in disease pathogenesis and repair. Functional experiments in this study showed that knockdown of CTSB enhanced endothelial cell proliferation, migration and tube formation ability, while overexpression had the opposite effect. These results suggested that CTSB acted as a negative regulator of angiogenesis and its abnormal expression exacerbates ischemia caused by PVD in the pathological context of DFU complicated with PVD. This regulatory role is particularly significant in the hyperglycemic microenvironment of diabetes. High glucose are found to impair endothelial function and inhibits vascular regeneration and repair^18^. High glucose can induce CTSB overexpression through hypoxia-inducible factor 1α and advanced glycation end products pathways^19^. Elevated CTSB further inhibits angiogenesis by degrading pro-angiogenic factors such as VEGF and basic fibroblast growth factor while suppressing endothelial cell survival and migration^20^. This mechanism explains why this study found that CTSB expression is associated with the severity of DFU complicated with PVD. Higher CTSB levels indicate suppressed vascular repair, exacerbating tissue ischemia and ulcer progression.

Comparison with existing biomarkers further highlights the unique value of CTSB. Although VEGF and angiopoietins have been proposed as markers for DFU-related angiogenesis, their diagnostic value is limited due to large expression fluctuations and weak correlation with disease stages^9^. In contrast, CTSB integrates multiple pathogenic pathways, reflecting both the hyperglycemic stress characteristics of diabetes^19^ and the impaired angiogenesis features of PVD^20^, thus serving as a more comprehensive indicator of the severity of DFU complicated with PVD. Its stability in serum and the detectable changes with Wagner grade observed in this study further enhance its potential for early diagnosis. Identifying elevated CTSB in early-stage DFU can prompt active vascular intervention to prevent progression to severe ulceration or amputation.

This study had certain limitations. Notably, the sensitivity and specificity of serum CTSB as a diagnostic biomarker for DFU complicated with PVD were not systematically evaluated due to the relatively small sample size. Furthermore, this study focused on clinical correlations and mechanistic links in the current design, which restricted definitive recommendations for its routine clinical use. Future research will expand multi-center clinical samples to perform receiver operating characteristic curve analysis, verifying its diagnostic efficacy in diverse DFU patients, and determining optimal cut-off values for distinguishing disease subtypes. Besides, in the context of evaluating CTSB as a serum biomarker, it is important to note that our study focused solely on protein levels measured via ELISA, without assessing CTSB RNA levels in serum. This is rooted in the biological nature of secreted proteins: CTSB is synthesized intracellularly, where its gene is transcribed into mRNA and translated into protein before being secreted into the extracellular space, including serum. Thus, mRNA primarily resides within the cells producing the protein, while serum contains the mature secreted protein. Serum itself is rich in nucleases, leading to rapid degradation of any cell-free RNA, resulting in extremely low, unstable levels that are technically challenging to detect reliably. However, this means our study lacks data on whether transcriptional regulation (reflected by RNA levels in source tissues) aligns with serum protein levels, which represents a limitation in fully characterizing CTSB’s expression dynamics. To address this, future studies could leverage diabetic mouse models of DFU complicated with PVD, where ethical constraints on accessing human tissue samples are absent, to measure CTSB mRNA levels in ulcerated skin tissues and correlate them with serum protein levels, thereby clarifying the link between transcriptional regulation and systemic protein abundance. Furthermore, this study only explored the regulatory mechanism of CTSB on angiogenesis through in vitro cell experiments, lacking animal model verification of its role in in vivo DFU wound repair. Thus, a diabetic mouse DFU model should be established to verify, through in vivo experiments, the promotional effect of CTSB knockdown or inhibition on wound vascular regeneration and healing in the future.

CTSB has sufficient basis as a potential serum biomarker for DFU complicated with PVD. Firstly, it has been confirmed as a secretory biomarker in vascular and inflammatory diseases. Secondly, it is clinically correlated with disease severity and core pathogenic factors. Thirdly, this study confirms that it participates in the key process of DFU healing by regulating angiogenesis. Thus, CTSB, as a serum detection biomarker, facilitates early diagnosis, monitors progression, and provides a basis for early intervention and individualized management of DFU complicated with PVD.

## Conclusion

Early PVD identification in DFU patients helps with early DFU treatment, increasing the likelihood of recovery and lowering the risk of sequelae. At the clinical level, single-cell transcriptome analysis found that CTSB was highly enriched in vascular endothelial cells of DFU, indicating CTSB was a potential target gene for DFU complicated with PVD. The immunohistochemistry and ELISA results of clinical samples revealed that CTSB was highly expressed in DFU complicated with PVD with the increase of the Wagner grade of DFU. Clinical correlation analysis indicated that the expression of CTSB is positively correlated with the clinical indicators of the combination of DFU and PVD. In vitro experiments, including tubule formation test, CCK8 assay and scratch test, proved that CTSB had angiogenesis function. These results indicated that CTSB was clinically associated with DFU complicated with PVD. CTSB was found to be detected in serum due to its property as a secretory protein in ELISA experiment of clinical samples. Therefore, this study suggests that CTSB can be used as a potential serum biomarker of DFU complicated with PVD. This study utilized a combined strategy of single-cell sequencing, molecular biology experiments, and clinical correlation analysis to explore the potential serum biomarkers of patients with the combination of DFU and PVD in a panoramic view. The identification of CTSB as a potential serum fluid biomarker of PVD in DFU patients is beneficial for early diagnosis, prognostic and progress monitoring, and treatment plan modification.

## Supplementary Information

Below is the link to the electronic supplementary material.

Supplementary Material 1

## Data Availability

The datasets analyzed during the current study are available in the GEO repository, and the persistent accession numbers to the datasets are GSE165816 and GSE223964.
