# Supplementary material for: Cathepsin B as a potential serum biomarker for early diagnosis and progression of diabetic foot ulcer complicated with peripheral vascular disease
Source: Sci Rep. 2025 Nov 27;15:42474. doi: 10.1038/s41598-025-26599-5 (PMC12660663; doi:10.1038/s41598-025-26599-5)
Supplement: Supplementary file 1 — Supplementary Material 1 [file 41598_2025_26599_MOESM1_ESM.pdf]

Supplementary Material

Table S1. The siRNA Sequences Targeting CTSB

Gene	Sense (5'-3')	Antisense (5'-3')
si-CTSB	CCGGGCACAACUUCUACAATT	UUGUAGAAGUUGUGCCCGGTT
si-NC	UUCUCCGAACGUGUCACGUTT	ACGUGACACGUUCGGAGAATT

si-CTSB: small interfering RNA targeting CTSB; si-NC: small interfering RNA negative control (si-NC).

Table S2. Quantitative Summary of IHC Results

Item	Groups	Mean	Standard deviation
Integrated density	non-DFU+PVD	5577360	1535893.01
	DFU-W3+PVD	20660780	2771721.32
	DFU-W4+PVD	28732465	581840.51
	DFU-W5+PVD	44873965	1847436.2
Proportion of positive area	non-DFU+PVD	2.36	0.65
	DFU-W3+PVD	8.73	1.17
	DFU-W4+PVD	12.15	0.25
	DFU-W5+PVD	19.67	0.81

non-DFU+PVD: non-diabetic foot ulcer combined with peripheral vascular disease (n = 3); DFU-W3+PVD: Wagner grade 3 diabetic foot ulcer combined with peripheral vascular disease (n = 3); DFU-W4+PVD: Wagner grade 4 diabetic foot ulcer combined with peripheral vascular disease (n = 3); DFU-W5+PVD: Wagner grade 5 diabetic foot ulcer combined with peripheral vascular disease (n = 3).

Table S3. Quantitative Summary of ELISA Results

Groups	Mean	Standard deviation
non-DFU+PVD	16.1	8.44
DFU-W3+PVD	33.93	15.33
DFU-W4+PVD	50.77	15.63
DFU-W5+PVD	74.78	21.71

non-DFU+PVD: non-diabetic foot ulcer combined with peripheral vascular disease (n = 36); DFU-W3+PVD: Wagner grade 3 diabetic foot ulcer combined with peripheral vascular disease (n = 26); DFU-W4+PVD: Wagner grade 4 diabetic foot ulcer combined with peripheral vascular disease (n = 21); DFU-W5+PVD: Wagner grade 5 diabetic foot ulcer combined with peripheral vascular disease (n = 10).
